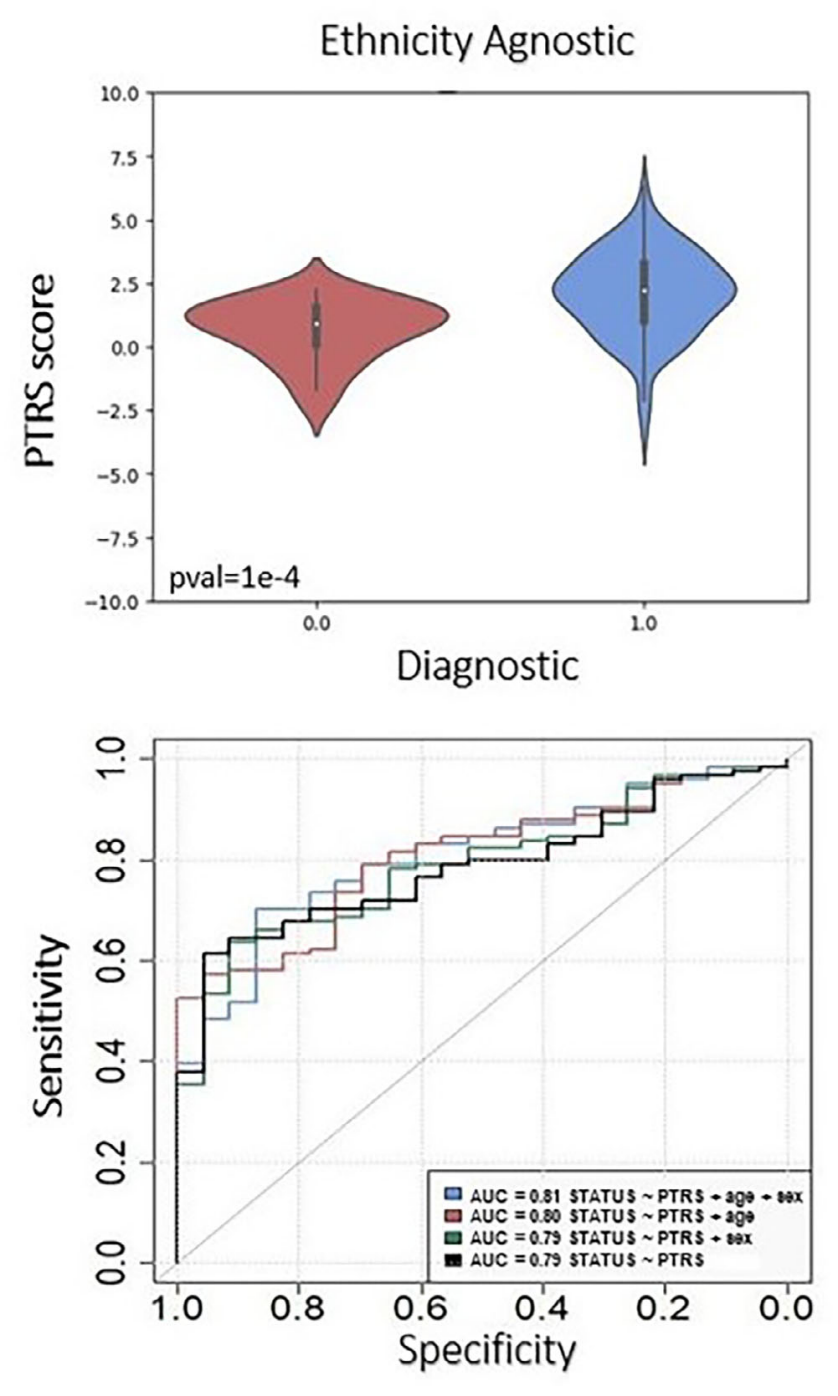# Polytranscriptomic risk score for Alzheimer Disease in a large diverse multi‐center brain bank study

**DOI:** 10.1002/alz.092971

**Published:** 2025-01-03

**Authors:** Basilio Cieza, Zikun Yang, Dolly Reyes‐Dumeyer, Annie J. Lee, Brittany N Dugger, Lee‐Way Jin, Melissa E. Murray, Dennis W. Dickson, Margaret A Pericak‐Vance, Jeffery M. Vance, Tatiana M. Foroud, Richard Mayeux, Giuseppe Tosto

**Affiliations:** ^1^ Columbia University, New York, NY USA; ^2^ The Taub Institute for Research on Alzheimer’s Disease and the Aging Brain, Vagelos College of Physicians & Surgeons, Columbia University, New York, NY USA; ^3^ Taub Institute for Research on Alzheimer’s Disease and The Aging Brain, Columbia University Medical Center, New York, NY USA; ^4^ University of California, Davis, Sacramento, CA USA; ^5^ University of California Davis Medical Center, Davis, CA USA; ^6^ Mayo Clinic, Jacksonville, FL USA; ^7^ Department of Neuroscience, Mayo Clinic, Jacksonville, FL USA; ^8^ 1501 NW 10th Avenue, Miami, FL USA; ^9^ John P. Hussman Institute for Human Genomics, Miami, FL USA; ^10^ Department of Neurology, Indiana University School of Medicine, Indianapolis, IN USA; ^11^ Department of Neurology, Vagelos College of Physicians & Surgeons, Columbia University, New York, NY USA

## Abstract

**Background:**

Alzheimer’s disease (AD) missing heritability remains extensive despite numerous genetic risk loci identified by genome‐wide association or sequencing studies. This has been attributed, at least partially, to mechanisms not currently investigated by traditional single‐marker/gene approaches. Polygenic Risk Scores (PRS) aggregate sparse genetic information across the genome to identify individual genetic risk profiles for disease prediction and patient risk stratification. Recent advancements have pivoted on innovative approaches utilizing OMICS data to construct such risk scores.

**Method:**

We employed a random forest algorithm to identify a list of gene candidates from bulk RNA sequencing data in prefrontal cortex from 565 AD brain samples (non‐Hispanic Whites, n = 399; Hispanics, n = 113; African American, n = 12) across six U.S. brain banks. Subsequently, we calculated their effect size on Braak staging using regression models to construct a polytranscriptomic risk score (PTRS). We employed two distinct models: “Ethnicity‐Agnostic” Model (randomly assigning samples to training and testing samples) and “Ethnicity‐Aware” Model (assigning NHW samples to training and Hispanics to testing sample). Analysis of variance and the receiver operating characteristics area under the curve (ROC AUC) was used to evaluate PTRS’s classification performances. We validated findings using the Religious Orders Study/Memory and Aging Project study (ROS/MAP, n = 1,095).

**Result:**

We found a significant difference in PTRS between samples with low vs. high Braak stages (≤4 vs. ≥5, p = 1*E‐04; **Figure 1 upper panel**). AUC was found to be 79‐81%, consistently in both Ethnicity‐Agnostic and Ethnicity‐Aware models (**Figure 1 lower panel)**. Finally, the PTRS in ROS/MAP yielded a similar classification performance (p = 2*E‐04, AUC = 77%).

**Conclusion:**

contrary to prior studies, we developed a PTRS with optimal transferability across ethnicities. This underscores the importance of developing novel tools to stratify and harmonize large brain repositories for AD.